# Perceived autonomy, family cohesion, and loneliness among community-dwelling older adults in China: a cross-sectional study

**DOI:** 10.3389/fpsyt.2026.1948241

**Published:** 2026-10-13

**Authors:** Jing Hua, Yuping Zhang, Yuanyuan Chen, Yanjie Liu, Jingfen Jin

**Affiliations:** Nursing Department, the Second Affiliated Hospital of Zhejiang University School of Medicine, Hangzhou, China

**Keywords:** autonomy, community-dwelling older adults, family cohesion, indirect association, loneliness

## Abstract

**Background:**

Loneliness is a significant public health concern among older adults and is associated with various adverse physical and psychological outcomes. Self-determination theory suggests that autonomy satisfaction may coexist with more voluntary engagement and emotional connectedness in family relationships. Few studies have examined the association between autonomy, family cohesion, and loneliness among Chinese community-dwelling older adults.

**Objective:**

This study examined the direct association between perceived autonomy and loneliness and the indirect association through family cohesion among community-dwelling older adults in China.

**Methods:**

A cross-sectional study was conducted among community-dwelling older adults in China. Data were collected using self-report questionnaires, including the Chinese version of the Perceived Autonomy Scale, the Family Adaptability and Cohesion Evaluation Scales II, and the UCLA Loneliness Scale. PROCESS Macro (model 4) for SPSS was used to examine the indirect association between perceived autonomy and loneliness through family cohesion while adjusting for covariates. The significance of the indirect association was tested using 5,000 bootstrap resamples with 95% confidence intervals.

**Results:**

Data from 447 community-dwelling older adults [age 75.24 ± 7.06 (range: 65-96) years] were analyzed. Perceived autonomy was positively correlated with family cohesion (r = 0.194, p < 0.001) and negatively correlated with loneliness (r = -0.369, p < 0.001); family cohesion was negatively correlated with loneliness (r = -0.373, p < 0.001). After controlling for the covariates, perceived autonomy was positively associated with family cohesion (B = 0.204, 95% CI [0.067, 0.342]), whereas perceived autonomy (B = -0.168, 95% CI [-0.218, -0.118]) and family cohesion (B = -0.094, 95% CI [-0.128, -0.060]) were negatively associated with loneliness. A significant indirect association between perceived autonomy and loneliness through family cohesion was observed (B = -0.019, BootSE = 0.008, 95% bootstrap CI [-0.036, -0.005]), accounting for approximately 10.26% of the total association.

**Conclusion:**

Among community-dwelling older adults in China, higher perceived autonomy was associated with lower loneliness, and a significant indirect association through family cohesion was observed. These findings suggest that perceived autonomy and family cohesion may be important correlates of psychological well-being and loneliness in later life. However, cross-sectional design limited the establishment of causality. Longitudinal and intervention studies are needed to determine whether approaches supporting older adults’ autonomy and family relationships are associated with subsequent changes in loneliness.

## 1 Introduction

### 1.1 Background

Population aging is a global phenomenon, with maintaining psychological well-being in later life emerging as a key priority (1). Loneliness, a distressing subjective experience arising from a perceived discrepancy between desired and actual social relationships (2), has emerged as a significant public health concern among older adults (3). Susanty et al.’s review estimated that approximately 26% of older adults worldwide experience loneliness, with a higher prevalence reported in Asia than in Europe and Oceania (4). China has the world’s largest older population and is undergoing rapid population changes, making loneliness a pressing but understudied issue (5). A recent national survey involving more than 220,000 Chinese older adults found that 36.6% experienced some degree of loneliness (6). A large number of studies have linked loneliness among older adults to adverse health outcomes, including increased risk of cardiovascular disease and mortality (7), cognitive decline (8), development of chronic diseases (9), and increased risks of hospitalization (10). Given the broad adverse consequences of loneliness in older adults (11), loneliness is a detrimental issue among older adults alongside population aging in China, which is important and worth exploring.

Previous study has shown that loneliness is not determined solely by objective social isolation or the number of social contacts (12). Even when exposed to similar social environments or levels of social contact, some older adults may perceive adequate emotional support and connectedness, whereas others may still experience profound loneliness (13). This suggests that loneliness is not merely determined by objective social factors, but also by individuals’ psychological needs, perceptions, and evaluations of interpersonal relationships (14). Understanding the determinants of older adults’ loneliness from a person-centered psychological perspective is therefore crucial. While existing studies were more focused on sociodemographic (15) (e.g., marital status, previous education level) and health-related (5) (e.g., physical or cognitive decline, being institutionalized) correlates of loneliness, the association of older adults’ basic psychological needs and cultural contexts with loneliness remains underexplored.

According to self-determination theory (SDT), autonomy is a fundamental psychological need, referred to as the experience of an individual’s sense of control and self-endorsement in daily life (16). Higher levels of perceived autonomy among older adults have been consistently linked to better psychological well-being and life satisfaction (17, 18). Existing evidence also shows that older adults who maintain autonomy are more likely to actively engage in social participation and maintain meaningful interpersonal relationships (19, 20). However, few studies have focused on the association between perceived autonomy and loneliness. A longitudinal study of 5,718 Swedish older adults reported that higher levels of autonomy were consistently associated with lower levels of loneliness, and that increases in autonomy over time were accompanied by decreases in loneliness (21). However, this national study was conducted primarily in a Western cultural context, and it remains unclear whether its findings can be generalized to older adults in Eastern populations.

Cultural context is particularly important when considering relevant factors of loneliness among older adults. Chinese culture is often characterized as collectivist and family-oriented, emphasizing filial piety, intergenerational reciprocity, and the family as the primary source of identity and support (22). Multigenerational co-residence and being cared for at home remain common among Chinese older adults, and they are expected to receive respect and care from their family members. In this context, family is often regarded as the primary source of emotional support, social connection, and interpersonal security for older adults with a collectivist cultural background (23). Hence, autonomy may be expressed in Chinese culture not through separation from the family but through maintaining dignity, communicating personal preferences, and participating in family decisions. Recent study have shown stronger family support has been associated with lower levels of loneliness among older adults (24). Family cohesion, which reflects emotional closeness, belonging, and connectedness among family members, is a particularly relevant aspect of the family environment (25). Unlike economic or functional support, family cohesion captures emotional bonding and interpersonal intimacy, which are more directly related to older adults’ subjective feelings and mental health (26). Gao et al.’s study reported family cohesion as an important cultural aspect of Chinese people, and reported it as a strong protective factor against negative mental outcomes among Chinese American older adults (27). The literature on the relationship between family cohesion and older adults’ loneliness is limited. Chokkanathan’s research conducted among 345 Asian older adults indicated that cohesive family environments were associated with lower loneliness and better psychological well-being (28). Hence, exploring the association between individual autonomy, family connectedness, and loneliness may place relatively greater emphasis on culturally appropriate understanding of loneliness among Chinese older adults.

### 1.2 Theoretical framework

This study was guided by self-determination theory (SDT) (29), which posits that autonomy, competence, and relatedness are three basic psychological needs essential for psychological adjustment and well-being (30). Although SDT was developed primarily in Western scholarship, emerging evidence from Chinese and cross-cultural samples indicates that psychological need satisfaction is also associated with older adults’ well-being in collectivist cultures. Yu et al.’s study investigated the associations between the three psychological needs and older adults’s subjective well-being guided by SDT (31). Furthermore, based on SDT, Tang et al. compared 510 Chinese and 170 French community-dwelling older adults and found that basic psychological need satisfaction promoted psychological well-being in both groups; and suggests the ways in which autonomy is expressed may vary across cultures (32). Among Chinese older adults, autonomy may coexist with family interdependence when family members respect older adults’ preferences and involve them in decisions that affect their lives.

From an SDT perspective, when individuals feel their autonomy is supported and respected, they are more capable of engaging in relationships authentically, which reflects in deeper emotional connections (33). Perceived autonomy may be associated with family relationship quality because older adults who feel that their preferences are respected may participate in family interactions more willingly and experience greater mutual understanding (34). Furthermore, SDT asserts that the frustration or deprivation of fundamental psychological needs is associated with psychological ill-being, such as loneliness (35). Within this framework, family cohesion may be positioned as a theoretical mediating factor because it captures the emotional bond, mutual support, and closeness that reflect the result of satisfaction of basic psychological needs (36), and may help explain the association between perceived autonomy and psychological well-being. Several previous studies have reported the mediating role of family cohesion among Chinese older adults, such as in the relationship between death anxiety and readiness toward advance care planning (37), and in the relationship between grandparenting proficiency and mental health (38). However, it remains underexplored whether family cohesion statistically mediates the association between perceived autonomy and loneliness among community-dwelling older adults in China.

### 1.3 Purpose and hypothesis

Based on SDT, this study aimed to investigate the associations among perceived autonomy, family cohesion, and loneliness, and examine whether family cohesion mediated the association between perceived autonomy and loneliness among community-dwelling older adults in China. The following hypotheses were addressed: 1) perceived autonomy would be negatively associated with loneliness (Hypothesis 1); 2) perceived autonomy would be positively associated with family cohesion (Hypothesis 2); 3) family cohesion would be negatively associated with loneliness (Hypothesis 3); 4) the association between perceived autonomy and loneliness would be indirectly linked through family cohesion (Hypothesis 4) among older adults. By clarifying those pathways, the present study provides insights for family- and community-based interventions.

## 2 Materials and methods

### 2.1 Study design

This cross-sectional study was conducted in Hangzhou, Zhejiang Province, China, from July to November 2025. Although the cross-sectional design limits the ability to infer causal relationships, it is appropriate for examining the associations among these variables and identifying potential relational pathways among community-dwelling older adults at a specific point in time (39). The study is in line with the Strengthening the Reporting of Observational Studies in Epidemiology (STROBE) checklist (**Supplementary File**).

### 2.2 Participants

The inclusion criteria were as follows: (a) aged 65 years or older; (b) no language communication barriers or severe cognitive impairment, ensuring the ability to complete the questionnaire; and (c) provision of informed consent and voluntary participation. Exclusion criteria included: (a) serious mental or physical illnesses; (b) inability to read or understand the questionnaire; and (c) unwillingness to cooperate with the survey.

### 2.3 Sample size

The minimum sample size was estimated using G*Power version 3.1.9.6 (40). Henning et al.’s longitudinal study was used only as contextual evidence suggesting that the association between autonomy and loneliness may be relatively modest (21); its unstandardized coefficient was not directly converted into the effect size used in G*Power for the current study because unstandardized coefficients are scale-dependent and derived from a different statistical design. Because G*Power does not directly estimate power for a bootstrap indirect effect, the calculation was based on the incremental contribution of one focal predictor in the most parameter-rich regression equation underlying PROCESS Model 4. As the sample-size calculation was conducted before data analysis, it was based on the maximum planned model, which included one independent variable (perceived autonomy), one mediator (family cohesion), and 11 covariates. Using a small effect size (f² = 0.05), α = 0.05, and a statistical power of 0.80, the minimum required sample size was 369 participants.

### 2.4 Data collection

A convenience sampling method was adopted. Researchers collaborated with official community workers and physicians from two community health service centers and their three affiliated service stations to recruit older adults who met the inclusion criteria. Then, eligible participants completed the paper-based questionnaires under the guidance of researchers. For participants with vision or literacy difficulties, researchers provided reading or clarification support without interfering with responses. Procedures were standardized, and all data were entered by two researchers to ensure accuracy.

### 2.5 Measurements

#### 2.5.1 Demographic characteristics

Based on previous evidence concerning factors associated with loneliness in older adults (5, 6, 41), the prespecified sociodemographic and health-related variables included age, sex, residence region, marital status, educational level, religion, living conditions, number of children, monthly income, self-rated health, and number of chronic diseases.

#### 2.5.2 Self-rated health

Self-rated health was assessed using a single-item measure that asked participants to evaluate their overall current health status. Responses were rated on a 5-point Likert scale ranging from 1 (“very poor”) to 5 (“very good”), with higher scores indicating better perceived health (42).

#### 2.5.3 Perceived autonomy

Perceived autonomy was measured using the Chinese version of the Perceived Enactment of Autonomy Scale, which was translated and revised by Wang et al. (43) based on the Taiwan version developed by Huang et al. (44). The scale consists of three dimensions: freedom of choice (8 items), individualism (9 items), and self-direction (4 items), with a total of 21 items. Each item is rated on a 4-point Likert scale ranging from “never” to “always,” scored from 1 to 4, respectively. The total score ranges from 21 to 84, with higher scores indicating higher levels of perceived autonomy. Wang et al. (43) adapted the Chinese version to assess perceived autonomy among older adults in mainland China, demonstrating good reliability, with a Cronbach’s alpha of 0.910 and a split-half reliability coefficient of 0.914; the adapted scale has subsequently been widely used among Chinese older adults (45). In this study, Cronbach’s alpha of the scale was 0.819, indicating good reliability.

#### 2.5.4 Family cohesion

Family cohesion was assessed using the Chinese version of the Family Adaptability and Cohesion Scale II, developed by Olson (46), translated and revised into Chinese by Phillips (47). This scale is used to assess two aspects of family function, including cohesion (e.g., emotional connection between family members) and adaptability (e.g., family system’s capabilities to overcome difficulties). In this study, only the subscale of the cohesion part was used. The cohesion subscale included 16 items, and each item was scored on a 5-point Likert scale. A higher score indicates better family cohesion. The Chinese version has been used as a reliable instrument to assess family functioning among older adults in both community and clinical settings (37). Cronbach’s alpha of the cohesion subscale in this study was 0.872.

#### 2.5.5 Loneliness

The UCLA-8 loneliness scale was adapted by Hays and DiMatteo (48) based on the UCLA-20 scale (University of California Los Angeles Loneliness Scale) (49). A 4-point scale (1 = never, 4 = always) was used to rate each item, with total scores ranging from 8 to 32. Items 3 (“I feel part of a group of friends”) and 6 (“I am an outgoing person”) were reverse-coded so that higher values represented greater loneliness. The UCLA-8 has previously been used among Chinese older adults and showed good internal consistency, with a Cronbach’s alpha of 0.915 (50). The Cronbach’s alpha in this study was 0.722, an acceptable internal consistency.

### 2.6 Data analysis

There was no missing data in the final analytic dataset. SPSS 31.0 (IBM, Chicago, IL, USA) and the PROCESS Macro for SPSS were used for statistical analysis. The two-tailed p-value of 0.05 was considered statistically significant. Based on the Kolmogorov–Smirnov test, the normality distribution was not confirmed. However, the skewness and kurtosis values of perceived autonomy (-0.77, 1.39), family cohesion (-0.36, 0.12), and loneliness (1.08, 0.99) were within ±2, so parametric analysis was performed. Correlations between age, self-rated health, and three main variables were estimated by Pearson correlation test. Independent sample t-tests and one-way analysis of variance (ANOVA) with Bonferroni correction were used to describe unadjusted differences in demographic variables in loneliness. Covariates were selected based on previous empirical evidence (5, 6) and theoretical relevance (32) to loneliness among older adults. Accordingly, the final model included age, sex, marital status, educational level, living condition, number of children, monthly income, self-rated health, and number of chronic diseases, representing relevant sociodemographic, family-related, socioeconomic, and health-related factors.

Hayes’ PROCESS Macro for SPSS (51) is widely used in health science research to test regression models with mediating effects by adjusting for covariates. PROCESS Model 4 was used to examine the direct association between perceived autonomy and loneliness and the indirect association through family cohesion. The statistical significance of the indirect association was evaluated using 5,000 bootstrap resamples and was considered significant when the 95% bootstrap confidence interval did not include zero.

### 2.6 Common method bias test

Since all study variables were collected through self-reported surveys, common method bias (CMB) is a potential concern. To mitigate the impact of CMB on the validity of the results, several procedural measures were implemented during the survey process, including balanced item ordering, anonymous questionnaire administration, and standardized measurement protocols. Additionally, Harman’s single-factor test was conducted to assess the extent of CMB. The results indicated that the first principal component accounted for 21.138% of the total variance, which is below the recommended threshold of 40%, suggesting that common method bias was not a significant issue in this study.

### 2.7 Ethical considerations

This study proceeded according to the principles of the Declaration of Helsinki and was approved by the Ethics Committee of the Second Affiliated Hospital of Zhejiang University School of Medicine (Approval No. 2025-1227). The participants were informed of their voluntary and anonymous participation. Completion and submission of the questionnaire were regarded as consent to participate. Upon completion of the questionnaire, participants were given a package of tissues (RMB 20 ≈ USD 2.5) for their participation.

## 3 Results

### 3.1 Participant characteristics

Overall, 462 questionnaires were distributed, 447 were included in the data analysis (effective response rate: 96.8%). Among the 447 participants (**Table 1**), there were 289 (64.7%) females and 158 (35.3%) males. The mean age was 75.24 ± 7.06 (range: 65–96) years. Moreover, 239 (53.5%) older adults lived only with their spouse, and 63 (14.1%) older adults lived alone. In addition, the majority (88.6%) of older adults had chronic diseases, and 51.0% reported two or more chronic diseases.

**Table 1:** Demographic characteristics of the study participants (n = 447).

Demographics	n (%)	Mean (SD)	Loneliness	
			t/F value	*p*
Sex			0.440^a^	0.660
Male	158 (35.3)	12.61 ± 3.77		
Female	289 (64.7)	12.45 ± 3.89		
Residence region			0.499^a^	0.618
Within Hangzhou	406 (90.8)	12.53 ± 3.85		
Outside Hangzhou	41 (9.2)	12.22 ± 3.88		
Marital status			-4.258^a^	< 0.001
Married	353 (79.0)	12.11 ± 3.52		
Widowed/single	94 (21.0)	13.98 ± 4.60		
Education level			3.026^b^	0.018
Primary school or below	129 (28.9)	13.39 ± 3.95		
Junior high school	187 (41.8)	12.13 ± 4.00		
Senior high school	80 (17.9)	11.96 ± 2.67		
College degree or above	44 (9.8)	12.18 ± 4.36		
Prefer not to answer	7 (1.6)	14.29 ± 3.40		
Religion			0.102^b^	0.904
None	357 (79.9)	12.52 ± 3.85		
Buddhism	72 (16.1)	12.56 ± 3.97		
Christianity	18 (4.0)	12.11 ± 3.34		
Living conditions			8.399^b^	< 0.001
Only with spouse	239 (53.5)	12.04 ± 3.61		
With child or whole family together	145 (32.4)	12.52 ± 3.59		
Alone	63 (14.1)	12.24 ± 4.73		
Number of children			2.878^b^	0.023
None	7 (1.6)	15.71 ± 5.56		
Single	296 (66.2)	12.40 ± 3.80		
2	107 (23.9)	13.04 ± 4.01		
3 or more	33 (7.4)	11.15 ± 2.90		
Prefer not to answer	4 (0.9)	12.00 ± 1.15		
Number of chronic diseases			3.934^b^	0.009
None	51 (11.4)	11.08 ± 3.16		
1	168 (37.6)	12.27 ± 3.60		
2	116 (26.0)	12.91 ± 4.23		
3 or more	112 (25.0)	13.10 ± 3.93		
Monthly income (RMB)			0.513^b^	0.674
Less than 3,000	58 (13.0)	12.40 ± 3.63		
3,000 ~ Less than 5,000	160 (35.8)	12.81 ± 3.88		
5,000 and above	227 (50.8)	12.32 ± 3.90		
Prefer not to answer	2 (0.4)	12.50 ± 0.71		

a: student’s t-test; b: one-way ANOVA with Bonferroni test.

SD, standard deviation.

### 3.2 Preliminary correlation analyses between the main variables

Older adults who were widowed or single reported significantly higher levels of loneliness than married participants (t = -4.258, *p* < 0.001). Significant differences in loneliness were also observed across education levels (F = 3.026, *p* = 0.018), living conditions (F = 8.399, *p* < 0.001), number of children (F = 2.878, *p* = 0.023), and number of chronic diseases (F = 3.934, *p* = 0.009). Specifically, older adults without children reported the highest loneliness scores, whereas those with three or more children showed the lowest loneliness levels. Participants without chronic diseases reported lower loneliness compared with those with multiple chronic diseases (**Table 1**).

**Table 2** presents the descriptive statistics and Pearson correlation tests of the main study variables. Perceived autonomy (r = 0.194, *p* < 0.001) was significantly positively associated with family cohesion and negatively associated with loneliness (r = -0.369, *p* < 0.001). Family cohesion was significantly negatively associated with loneliness (r = -0.373, *p* < 0.001). Age (r = 0.128, *p* < 0.01) and self-rated health (r = -0.214, *p* < 0.001) were statistically associated with loneliness. Moreover, multicollinearity diagnostics were examined for all variables included in the model. No problematic multicollinearity was detected, as all variance inflation factor (VIF) values were below 3 and all tolerance values were above 0.10 (**Supplementary Table 1**).

**Table 2:** Mean scores and Pearson correlation coefficients of main variables (n = 447).

Variables	Mean (SD)	1	2	3	4
1. Age	75.24 ± 7.06	1			
2. Self-rated health	3.15 ± 0.82	0.126**	1		
3. Family cohesion	67.48 ± 10.71	-0.087	0.203***	1	
4. Perceived autonomy	71.58 ± 6.88	-0.251***	0.296***	0.194***	
5. Loneliness	12.51 ± 3.85	0.128**	-0.214***	-0.373***	-0.369***

**p < 0.01; ***p < 0.001.

SD, standard deviation.

### 3.3 Direct and indirect effects among the main study variables

**Figure 1** illustrates the standardized path coefficients for mediation models among perceived autonomy, and **Table 3** presents the direct association between perceived autonomy, family cohesion, and loneliness. After adjusting for covariates, perceived autonomy was positively associated with family cohesion (β = 0.131; B = 0.204, 95% CI [0.067, 0.342], *p* = 0.004) and negatively associated with loneliness (β = -0.301; B = -0.168, 95% CI [-0.218, -0.118], *p* < 0.001). Family cohesion was also negatively associated with loneliness (β = -0.261; B = -0.094, 95% CI [-0.128, -0.060], *p* < 0.001).

**Figure 1:**
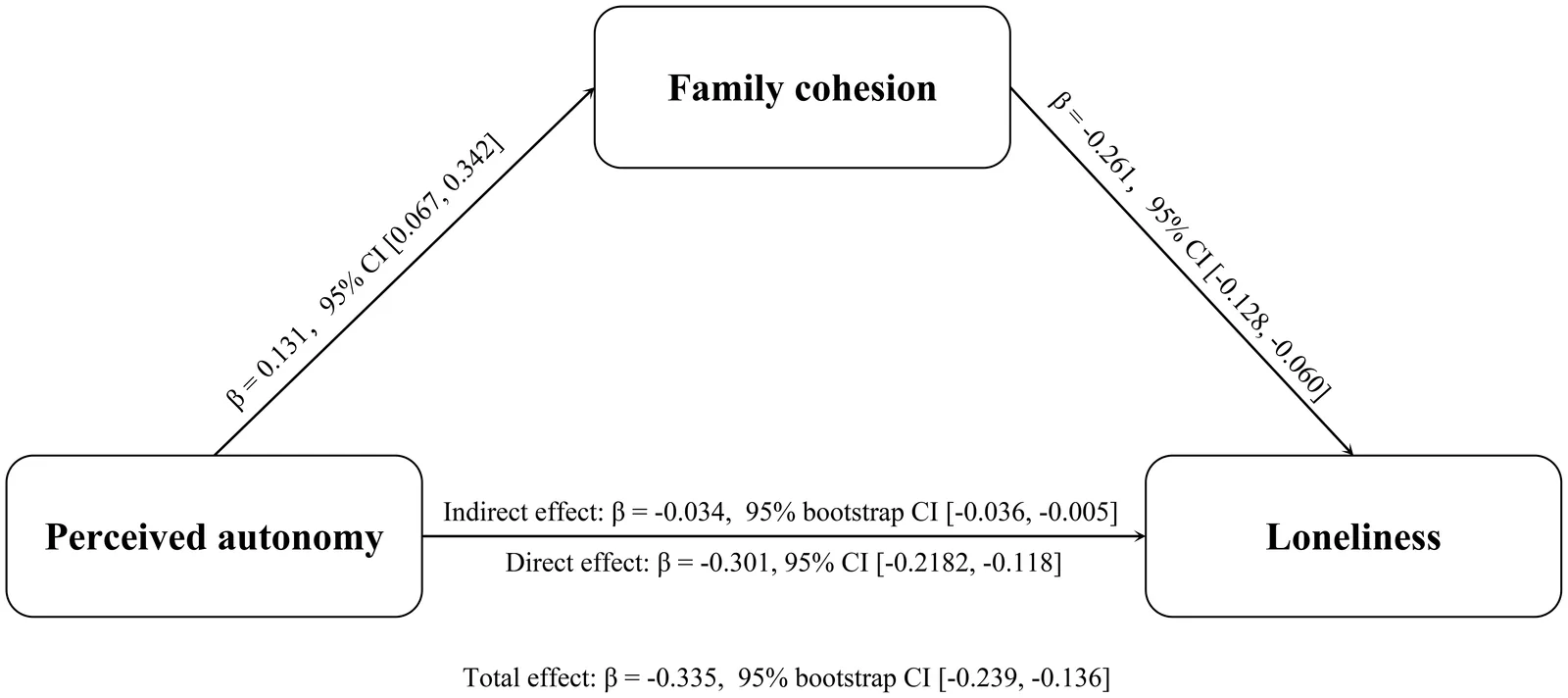
Indirect-association model of perceived autonomy, family cohesion, and loneliness. Single-headed arrows represent the hypothesized statistical paths and should not be interpreted as evidence of temporal or causal relationships. Age, sex, marital status, education level, living condition, number of children, monthly income, self-rated health, and number of chronic diseases were included as covariates in the statistical analysis (full model results with covariates were reported in **Table 3**).

**Table 3:** Direct associations among perceived autonomy, family cohesion, and loneliness (PROCESS Model 4, n = 447).

Variables	Outcome variable: Family cohesion						Outcome variable: Loneliness					
	β	B	SE	*p*	LLCI	ULCI	β	B	SE	*p*	LLCI	ULCI
Perceived autonomy	0.131	0.204	0.070	0.004	0.067	0.342	-0.301	-0.168	0.026	< 0.001	-0.218	-0.118
Family cohesion							-0.261	-0.094	0.017	< 0.001	-0.128	-0.060
Age	0.027	0.041	0.078	0.603	-0.113	0.195	0.025	0.014	0.028	0.626	-0.042	0.069
Sex (ref. male)	0.044	0.986	0.983	0.316	-0.947	2.919	-0.067	-0.535	0.355	0.133	-1.232	0.163
Marital status (ref. married)	-0.086	-2.248	1.511	0.138	-5.218	0.722	0.123	1.157	0.546	0.035	0.083	2.231
Educational level	0.112	1.209	0.473	0.011	0.280	2.138	-0.024	-0.094	0.172	0.583	-0.432	0.243
Living conditions (ref. Only With Spouse)												
With child or whole family together	-0.392	-12.067	1.770	< 0.001	-15.546	-8.587	-0.020	-0.215	0.672	0.749	-1.535	1.105
Alone	-0.115	-2.630	1.084	0.016	-4.760	-0.500	-0.018	-0.146	0.394	0.710	-0.920	0.627
Number of children	0.104	1.613	0.722	0.026	0.194	3.032	-0.119	-0.666	0.262	0.011	-1.180	-0.151
Monthly income	0.010	0.147	0.699	0.834	-1.227	1.521	0.019	0.104	0.252	0.680	-0.391	0.600
Self-rated health	0.131	1.717	0.599	0.004	0.539	2.895	-0.055	-0.260	0.218	0.234	-0.689	0.169
Number of chronic diseases	-0.027	-0.292	0.490	0.552	-1.255	0.672	0.061	0.241	0.177	0.175	-0.107	0.588
Constant		43.609	8.284	< 0.001	27.328	59.891		30.45	3.081	< 0.001	24.394	36.506
**Model R^2^**	**0.267**						**0.263**					

β, standardized regression coefficient; B, unstandardized regression coefficient; SE, standard error; LLCI, lower limit of the 95% confidence interval; ULCI, upper limit of the 95% confidence interval. Ref, reference categories. Model R²: the proportion of variance in the outcome variable explained by the variables included in the model.

Bootstrap analysis with 5,000 resamples showed that the total effect of perceived autonomy on loneliness was statistically significant (β = -0.335; B = -0.187, SE = 0.026, 95% CI [-0.239, -0.136], *p* < 0.001) and there was a statistically significant indirect association between perceived autonomy and loneliness through family cohesion (indirect effect: B = -0.019, BootSE = 0.008, 95% bootstrap CI [-0.036, -0.005]; completely standardized indirect effect: β = -0.034, 95% bootstrap CI [-0.064, -0.009]). The indirect association accounted for approximately 10.26% of the total association, whereas the remaining 89.74% was attributable to the direct association. Therefore, Hypotheses 1–4 were supported.

## 4 Discussion

Guided by self-determination theory, this study examined the associations among perceived autonomy, family cohesion, and loneliness in community-dwelling older adults in China. The findings indicated that greater perceived autonomy was associated with lower loneliness, both directly and indirectly through higher family cohesion; all proposed hypotheses were supported. However, given the cross-sectional design, this indirect association should not be interpreted as evidence of temporal ordering or causal mediation.

The present study found that greater perceived autonomy was associated with lower loneliness among community-dwelling older adults in China. This finding is consistent with, although not directly comparable to, previous evidence from different Chinese and international studies. A nationwide longitudinal study in China found that limited financial decision-making power was associated with greater loneliness among older adults (52), while qualitative evidence from Hong Kong highlighted the importance of autonomy in maintaining dignity and well-being in later life (53). Together, these findings suggest that participation in household decision-making may represent a culturally salient expression of autonomy by conveying respect for older adults’ preferences and recognition of their family roles. A Swedish longitudinal study similarly reported that higher autonomy was associated with lower loneliness over four years (21). However, that study included adults aged 60–66 years and employed longitudinal latent curve modeling, whereas the present study used a cross-sectional design and included community-dwelling older adults over 65 years old in China. Rather than reflecting a simple distinction between individual independence in Western cultures and family interdependence in Chinese culture, autonomy may be expressed in culturally and relationally specific ways. For Chinese older adults, it may include both personal choice and meaningful participation in family decisions and roles (32). The present study, therefore, extends previous evidence to Chinese community-dwelling older adults while indicating that the association between autonomy and loneliness should be understood within its cultural and relational context.

The present study also found that perceived autonomy was positively associated with family cohesion. From the perspectives of SDT and Chinese family-oriented values, this finding suggests that autonomy among older adults should not be understood as complete independence or separation from family members (54). Instead, the experience of autonomy may coexist with emotional interdependence and supportive family relationships (31). Older adults who perceive that their preferences are respected may also report greater willingness to participate in family interactions, communicate their needs, and maintain meaningful family roles, characteristics that may coincide with greater mutual understanding and emotional closeness (28, 55). The contribution of the present finding is therefore not to demonstrate that autonomy increases family cohesion, but to show that perceived autonomy and family cohesion are positively related rather than mutually incompatible within a family-oriented cultural context.

The present study further identified a significant indirect association between perceived autonomy and loneliness through family cohesion, accounting for approximately 10.26% of the total association. This finding is broadly consistent with previous research emphasizing the relevance of family relationships to loneliness among Chinese older adults. For example, Lu and Zhang found that stronger family social capital was associated with lower social and emotional loneliness among community-dwelling older adults in urban China (56), while Zhao et al. reported that intergenerational reciprocity, participation in family decision-making, and continued contributions to adult children were associated with lower loneliness (57). Although family social capital and intergenerational reciprocity are conceptually distinct from family cohesion, these studies collectively suggest that the emotional and relational quality of family life may be particularly relevant to loneliness in the Chinese cultural context. The present study adds to this literature by examining perceived autonomy and family cohesion simultaneously within the same analytical model. From an SDT perspective situated within a family-oriented cultural context, one possible interpretation is that perceived autonomy may coexist with respectful family involvement and stronger emotional connectedness, while family cohesion may reflect the belonging, emotional support, and relational security associated with lower loneliness (24, 58). However, this statistical indirect association should not be interpreted as evidence that family cohesion is an established causal mechanism. Reciprocal relationships and unmeasured family or social factors may also account for the observed pattern. Moreover, the relatively small magnitude of the indirect association and the remaining direct association indicate that family cohesion is likely to represent only one of several relational and individual factors involved in the association between perceived autonomy and loneliness.

## 5 Implications for practice

### 5.1 Supporting autonomy among community-dwelling older adults

The negative association between perceived autonomy and loneliness highlights the potential relevance of preserving older adults’ sense of volition, control, and participation in everyday life. Community health professionals, geriatric mental health practitioners, family caregivers, and policymakers may therefore consider incorporating autonomy-supportive practices into broader programs designed to promote older adults’ psychological well-being. In community and family settings, older adults could be encouraged to express their preferences and participate in decisions concerning health care, daily routines, and even family matters.

### 5.2 Considering family cohesion in loneliness-related services

The observed indirect association through family cohesion suggests potential directions for prevention and mental health promotion among community-dwelling older adults. From a family perspective, the findings highlight the importance of respecting older adults’ preferences, involving them in family decision-making, and recognizing behaviors that may unintentionally restrict their autonomy. These family-oriented approaches may support older adults’ perceived autonomy, family cohesion, and psychological well-being.

## 6 Limitations and further research

This study has several limitations that also inform directions for future research. First, the cross-sectional design limits causal inferences regarding the relationships among perceived autonomy, family cohesion, and loneliness, and the observed indirect association through family cohesion should be interpreted as a statistical indirect association rather than evidence of a causal mechanism. Future longitudinal or prospective studies with multiple assessment waves, such as cross-lagged panel or longitudinal mediation models, are needed to clarify the causal relationship of these relationships. Second, participants were recruited from a limited geographic area in China using non-probability sampling, which may have introduced selection bias and restricted generalizability to older adults in other regions, cultural contexts, and socioeconomic circumstances. Future studies should therefore use probability sampling and recruit more diverse and representative samples, including older adults with varied socioeconomic and cultural backgrounds. Third, all variables were assessed through self-report measures, which may introduce common method variance bias and recall bias, potentially attenuating the observed associations. Future research could combine self-report measures with ecological momentary assessment or daily diaries of loneliness, structured interviews, or behavioral or observational measures of autonomy task and decision-making to reduce common method variance and recall bias. Fourth, although several demographic covariates were adjusted in the final analysis, other potentially relevant factors, such as physical functioning, social participation, and community support, were not measured; these factors may influence the indirect association between perceived autonomy and loneliness through family cohesion. Future studies should include a broader range of psychosocial and health-related variables to reduce residual confounding and better understand the factors associated with loneliness among older adults.

## 7 Conclusion

This study examined the association between perceived autonomy and loneliness among community-dwelling older adults, and a significant indirect association through family cohesion was observed. However, given the cross-sectional design, the result should be interpreted as an indirect association rather than as evidence of causality. The findings highlight the potential relevance of respecting older adults’ preferences and involving them in family decisions. However, longitudinal and intervention studies are required before these approaches can be recommended as effective strategies for preventing loneliness.

## Data Availability

The raw data supporting the conclusions of this article will be made available by the authors, without undue reservation.
