# Supplementary material for: Perceived autonomy, family cohesion, and loneliness among community-dwelling older adults in China: a cross-sectional study
Source: Front Psychiatry. 2026 Oct 13;17:1948241. doi: 10.3389/fpsyt.2026.1948241 (PMC13710357; doi:10.3389/fpsyt.2026.1948241)
Supplement: Supplementary file 1 [file Table1.docx]

**Supplementary Table 1. linear regression of Loneliness (n=447)**

| **Variables** | **β** | **B** | **SE** | ***p*** | **95%CI** | | **Collinearity** | |
| --- | --- | --- | --- | --- | --- | --- | --- | --- |
|  |  |  |  |  | **LLCI** | **ULCI** | **Tolerance** | **VIF** |
| Constant |  | 30.45 | 3.081 | <0.001 | 24.394 | 36.506 |  |  |
| Perceived Autonomy | -0.300 | -0.168 | 0.026 | <0.001 | -0.218 | -0.118 | 0.816 | 1.225 |
| Family cohesion | -0.261 | -0.094 | 0.017 | <0.001 | -0.128 | -0.06 | 0.733 | 1.365 |
| Age | 0.025 | 0.014 | 0.028 | 0.626 | -0.042 | 0.069 | 0.631 | 1.584 |
| Sex (ref. male) | -0.067 | -0.535 | 0.355 | 0.133 | -1.232 | 0.163 | 0.871 | 1.149 |
| Education level | -0.024 | -0.094 | 0.172 | 0.583 | -0.432 | 0.243 | 0.864 | 1.157 |
| Marital status (ref. married) | 0.123 | 1.157 | 0.546 | 0.035 | 0.083 | 2.231 | 0.506 | 1.976 |
| Living condition (ref. Only With Spouse) |  |  |  |  |  |  |  |  |
| With child or whole family together | -0.019 | -0.215 | 0.672 | 0.749 | -1.535 | 1.105 | 0.459 | 2.178 |
| Alone | -0.018 | -0.146 | 0.394 | 0.710 | -0.92 | 0.627 | 0.739 | 1.353 |
| Number of children | -0.119 | -0.666 | 0.262 | 0.011 | -1.18 | -0.151 | 0.775 | 1.291 |
| Monthly income | 0.019 | 0.104 | 0.252 | 0.680 | -0.391 | 0.600 | 0.780 | 1.281 |
| Self-rated health | -0.055 | -0.260 | 0.218 | 0.234 | -0.689 | 0.169 | 0.795 | 1.258 |
| Number of chronic diseases | 0.061 | 0.241 | 0.177 | 0.175 | -0.107 | 0.588 | 0.837 | 1.195 |
| Abbreviation: LLCl,Lower level confidence interval;ULCl,Upper level confidence interval; VIF, Variance Inflation Factor.  Linear regression analysis (enter): R^2^ = 0.262; F= 12.870, *p* < 0.001; Durbin-Watson value = 2.070 | | | | | | | | |
